# A High-Throughput Chemical Screen in *DJ-1β* Mutant Flies Identifies Zaprinast as a Potential Parkinson’s Disease Treatment

**DOI:** 10.1007/s13311-021-01134-2

**Published:** 2021-10-25

**Authors:** Francisco José Sanz, Cristina Solana-Manrique, Josema Torres, Esther Masiá, María J. Vicent, Nuria Paricio

**Affiliations:** 1 Departamento de Genética, Facultad CC Biológicas, Universidad de Valencia, 46100 Burjassot, Spain; 2 Instituto Universitario de Biotecnología Y Biomedicina (BIOTECMED), Universidad de Valencia, 46100 Burjassot, Spain; 3 Departamento de Biología Celular, Biología Funcional Y Antropología Física, Facultad CC Biológicas, Universidad de Valencia, 46100 Burjassot, Spain; 4 Polymer Therapeutics Lab and Screening Platform, Centro de Investigación Príncipe Felipe, 46012 Valencia, Spain

**Keywords:** *Drosophila*, Parkinson’s disease, Zaprinast, High-throughput screening, Phosphodiesterase inhibitor, GPR35 agonist

## Abstract

Dopamine replacement represents the standard therapy for Parkinson’s disease (PD), a common, chronic, and incurable neurological disorder; however, this approach only treats the symptoms of this devastating disease. In the search for novel disease-modifying therapies that target other relevant molecular and cellular mechanisms, *Drosophila* has emerged as a valuable tool to study neurodegenerative diseases due to the presence of a complex central nervous system, the blood–brain barrier, and a similar neurotransmitter profile to humans. Human PD-related genes also display conservation in flies; *DJ-1β* is the fly ortholog of *DJ-1*, a gene for which mutations prompt early-onset recessive PD. Interestingly, flies mutant for *DJ-1β* exhibit PD-related phenotypes, including motor defects, high oxidative stress (OS) levels and metabolic alterations. To identify novel therapies for PD, we performed an in vivo high-throughput screening assay using *DJ-1β* mutant flies and compounds from the Prestwick® chemical library. Drugs that improved motor performance in *DJ-1ß* mutant flies were validated in *DJ-1*-deficient human neural-like cells, revealing that zaprinast displayed the most significant ability to suppress OS-induced cell death. Zaprinast inhibits phosphodiesterases and activates GPR35, an orphan G-protein-coupled receptor not previously associated with PD. We found that zaprinast exerts its beneficial effect in both fly and human PD models through several disease-modifying mechanisms, including reduced OS levels, attenuated apoptosis, increased mitochondrial viability, and enhanced glycolysis. Therefore, our results support zaprinast as a potential therapeutic for PD in future clinical trials.

**Supplementary Information:**

The online version contains supplementary material available at 10.1007/s13311-021-01134-2.

## Introduction

Parkinson’s disease (PD), a progressive neurodegenerative movement disorder [1], is characterized by resting tremor, muscular rigidity, bradykinesia postural instability, and other non-motor symptoms that worsen patients’ quality of life [2]. The appearance of these symptoms is caused by the selective loss of dopaminergic neurons in the substantia nigra pars compacta, which leads to a reduction in dopamine levels in the striatum [3], and the formation of intracellular protein aggregates called Lewy bodies in neurons [4]. Additional factors associated with PD progression include elevated oxidative stress (OS) levels, mitochondrial dysfunction, and metabolic alterations [5, 6]. Striatal dopamine replacement using dopamine precursors, dopamine agonists, or inhibitors of dopamine metabolism [7] currently represents the most commonly employed treatment for PD; however, this therapeutic approach only treats symptoms during early-stage disease and does not modify the underlying disease in the long term. Furthermore, the efficacy of this treatment strategy decreases after long-term administration, and it cannot counteract or delay PD progression. Therefore, we require novel therapies that target other disease-related molecular and cellular mechanisms in the hope of modifying the underlying disease [8, 9]. In this sense, multiple studies have evaluated compounds whose mechanisms of action may impact calcium transport, OS levels, mitochondrial function, autophagy, or energy metabolism as potential PD treatments [7, 10, 11]. Notably, the current PD drug development pipeline, based on ongoing clinical trials, involves therapeutics that treat both symptoms and the underlying disease [12].

Although most PD cases are sporadic and involve the contribution of environmental factors and aging, there also exist familial cases caused by mutations in specific genes [13]. Studies have identified clear links between sporadic and familial PD cases, which are clinically and pathologically indistinguishable except for age at onset [14]. Functional studies of genes involved in familial PD have led to a better understanding of the mechanisms and molecular pathways underlying PD pathophysiology that may govern/influence progressive neurodegeneration [15–17]. Mutations in one such gene, *DJ-1* (also known as *PARK7*), associate with an early-onset recessive form of Parkinsonism [18]. *DJ-1* was initially described as an oncogene, but additional functions for the DJ-1 protein include roles as an antioxidant via free-radical scavenging, a transcriptional regulator of antioxidant genes, a redox-dependent molecular chaperone, a modulator of mitochondrial function, a deglycase, and as a factor involved in proteolysis and metabolism [19–22]. Also, a study encountered an over-oxidized and inactive form of the DJ-1 protein in brains of sporadic PD patients [23], strongly suggesting that results obtained in animal and cell models of familial PD based on loss of *DJ-1* function may also have relevance to the sporadic form of the disease [24].

*Drosophila* has recently emerged as an essential tool in the study of neurodegenerative diseases due to the presence of a complex central nervous system (CNS), a blood–brain barrier (BBB), and similar neurotransmitters to humans [25–27]. Furthermore, more than 70% of human disease-related genes display conservation in flies [28], including orthologs of genes involved in familial PD cases (e.g., *DJ-1*, *PINK1*, *PRKN*, or *LRRK2*) [29]. Flies harboring mutations in such genes exhibit PD-related phenotypes and have allowed the identification of potentially pathogenic mechanisms and modifiers of PD pathology through genetic or pharmacological approaches [30]. Our previous studies revealed that flies carrying mutations in *DJ-1β* (*Drosophila* ortholog of the human *DJ-1* gene) exhibited shortened lifespans, motor defects, high OS levels, and hypersensitivity to OS-inducing toxins [31, 32]. Interestingly, we also discovered that supplementation with antioxidant compounds efficiently suppressed some of these phenotypes, thereby confirming *Drosophila* as an amenable model organism to identify and validate new drugs with therapeutic potential in PD patients [31–33]. Supporting this assumption, we subsequently performed a pilot screen to evaluate the effect of antioxidant, anti-inflammatory, and neuroprotective compounds in *DJ-1β* mutant flies. Excitingly, selected compounds that attenuated motor defects associated with loss of *DJ-1β* function also increased the viability of *DJ-1*-deficient human neuroblastoma cells subjected to OS conditions [34], thereby supporting the translatability of pharmacological studies carried out in *Drosophila*. The conservation of the BBB in flies increases the probability of encountering novel and relevant therapeutic compounds to treat PD and other human diseases [27, 35].

The present study aimed to identify drugs as novel treatment options for PD through an in vivo high-throughput screening (HTS) assay in a *Drosophila* PD model (*DJ-1β* inactivating mutation) using compounds from the Prestwick® Chemical Library (PCL). We identified drugs that attenuated motor defects in *DJ-1β* mutant flies and then further evaluated the potential neuroprotective effect of candidates in *DJ-1*-deficient human neuron-like cells. Among the compounds identified, zaprinast (ZAP) treatment displayed the most significant reduction in OS-induced cell death. We found that ZAP suppressed multiple disease phenotypes in fly and human cell PD models based on *DJ-1* deficiency, and exhibited disease-relevant mechanisms of action. Thus, ZAP covers various aspects of translational cross-species and multi-phenotype modeling, as recommended to improve drug discovery in neurodegenerative diseases [36]. Taken together, our results support ZAP as a potentially interesting therapy for PD that could exert beneficial effects in future clinical trials.

## Material and Methods

### *Drosophila* Stocks

Fly stocks employed in this study were the *DJ-1β*^*ex54*^ strain (referred to as *DJ-1β*) from the J. Chung laboratory [37] and the *park*^*25*^ strain (referred to as *park*) from the A. J. Whitworth laboratory [38]. Stocks and fly crosses were cultured using standard *Drosophila* feed at 25 ºC unless otherwise indicated.

#### Drug Treatment and Climbing Assays

The effect of the 1120 compounds from the PCL, which are dissolved in 100% dimethyl sulfoxide (DMSO) at a final concentration of 5 mM, on the locomotor ability of *DJ-1β* mutant flies was evaluated. To do this, 40 L2 stage larvae were cultured in tubes (100 × 16 mm, SARSTEDT) with 1 ml of standard food containing 0.2% DMSO or supplemented with each compound at a final concentration of 10 µM. After eclosion, adult male and females flies were transferred to new tubes, and a climbing assay performed five days later using a protocol adapted from a previous study [34]. Briefly, flies were divided into 2–4 groups, depending on the amount of individuals hatching in that period. Subsequently, each group of flies was transferred to graduated plastic tubes, acclimated for 1 min, gently tapped down to the tube bottom, and allowed to climb for 10 s. This process was recorded and repeated four times for each group of flies. Climbing ability was determined as the average of the height reached by each group of flies. Compounds that improved locolomotor activity of flies with a *P* value below 0.05 were selected as positive candidates. All drugs were screened blindly. For further experiments, a new batch of zaprinast was obtained (Santa Cruz Biotechnology) and a 100 mM stock dissolved in DMSO was prepared.

We used a protocol adapted from a previous study for climbing assays in *park* mutants [38]. Briefly, 5 tubes (75 × 23.5 mm, SARSTEDT) with 80 L2 stage larvae each were cultured with 2.5 ml of standard food containing 0.1% DMSO or supplemented with 10 µM zaprinast. After hatching, homozygous *park* male flies were transferred to new tubes, and a climbing assay was performed 3 to 4 days later. Briefly, groups of 10–15 flies were acclimated for 1 min in new vials, gently tapped down to the tube bottom, and allowed to climb. We counted the number of flies that crossed a line drawn at 7 cm from the vial's bottom in 10 s. This process was recorded and repeated three times for each group of flies.

#### Cell Culture and Drug Treatment

*DJ-1-*deficient and *pLKO.1* control SH-SY5Y neuron-like cells previously generated by our laboratory [34] were cultured in Dulbecco’s Modified Eagle Medium/Nutrient Mixture F-12 (DMEM/F-12) supplemented with 10% fetal bovine serum, 1% non-essential amino acids, and 100 mg/ml penicillin/streptomycin at 37 °C and 5% CO_2_. All cell culture materials were purchased from Biowest. Viability of cells treated with candidate compounds, the GPR35 antagonist CID2745687 and DMSO as vehicle was evaluated using an MTT (3-(4, 5-dimethylthiazol-2-yl)-2–5-diphenyltetrazolium bromide) assay, as previously described [22]. To determine whether CID2745687 was able to interfere with the neuroprotective effect of zaprinast, cells were pretreated for 2 h with different concentrations of the compound before the addition of zaprinast. Next, viability assays were carried out as described [22]

#### Quantification of Protein Carbonyl Group Formation and H_2_O_2_ Levels

Protein carbonylation and H_2_O_2_ levels were measured in 5-day-old *DJ-1β* mutant flies that were treated with the vehicle (0.1% DMSO) as control or treated with 10 μM zaprinast. Protein carbonyl groups were measured in fly extracts using 2,4-dinitrophenyl hydrazine derivatization in 96-well plates (Greiner 96-well plate, polypropylene) as previously described [22]. H_2_O_2_ levels were measured using the Amplex Red Hydrogen Peroxide/Peroxidase Assay Kit (Invitrogen) in fly extracts as previously described [34]. All experiments were carried out using three biological replicates and three technical replicates for each sample.

#### Western Blotting

Protein extraction and Western blots of *pLKO.1* and *DJ-1*-deficient SH-SY5Y cells treated with 1 µM zaprinast and vehicle (0.1% DMSO) under OS conditions were carried out as previously described [22]. The primary antibodies used were anti-Akt, anti-phospho-Akt (Ser473), anti-JNK, and anti-phospho-JNK (Thr183/Tyr185) (1:1000, Cell Signaling). Secondary antibodies used were anti-rabbit or anti-mouse HRP-conjugated (1:5000, Sigma). Quantifications of protein levels were performed with an ImageQuant™ LAS 4000mini Biomolecular Imager (GE Healthcare), and images were analyzed with ImageJ software (NIH).

#### Mitochondrial Viability

Mitochondrial viability assays were performed using the MitoTracker™ Red FM (Invitrogen) fluorescence dye in *DJ-1-*deficient and control SH-SY5Y cells. Briefly, 50 × 10^4^ cells were seeded on glass coverslips in P60 cell culture dishes and incubated overnight. Next, the cell culture medium was removed, and fresh medium supplemented with 1 μM zaprinast or 0.1% DMSO was added and cells incubated for 24 h. Subsequently, the cell culture medium was removed, and cells were incubated in 60 nM Mitotracker™ Red FM diluted in incomplete cell culture medium for 30 min. Finally, cells were washed three times with PBS, and coverslips were mounted onto microscope slides with Vectashield (mounting medium) with DAPI (Vector Laboratories). Fluorescence images were acquired using fluorescence microscopy (Leica DMI3000 B) and analyzed with ImageJ software (NIH).

#### Enzymatic Assays

The enzymatic activities of enolase (Eno; EC 4.2.1.11), phosphofructokinase (Pfk; EC 2.7.1.11), pyruvate kinase (Pk; EC 2.7.1.40), and hexokinase (Hk; EC 2.7.1.1) were measured using coupled enzymatic assays in extracts of *pLKO.1* and *DJ-1*-deficient SH-SY5Y cells treated with 1 µM zaprinast or vehicle (0.1% DMSO) for 24 h as previously described [22]. All experiments were performed in triplicate.

#### Quantification of ATP Levels

ATP levels in SH-SY5Y cells were measured using the ATP Determination Kit (Invitrogen). One day prior to the assay, 10,000 *pLKO.1* or *DJ-1*-deficient cells were seeded in a white 96-well plate and incubated for 24 h with 1 µM zaprinast or with 0.1% DMSO as vehicle medium. Subsequently, they were incubated with 50 µM of H_2_O_2_ for 3 h, and later 100 µl of the ATP assay mix was added. Luminescence intensity was measured using an Infinite 200 PRO reader (Tecan). An MTT assay was also performed in order to calculate cell viability. All experiments were performed in triplicate and results are expressed as relative luminescence intensity per cell viability.

#### Statistical Analyses

The significance of differences between means was assessed using a *t*-test when two experimental groups were analyzed. In experiments in which more than two experimental groups were used, the statistical analysis was made using the ANOVA test and Tukey’s post hoc test. Differences were considered significant when *P* < 0.05. Data are expressed as means ± standard deviation (s.d.).

## Results

### A High-Throughput Chemical Screen in DJ-1β Mutant Flies and Validation in DJ-1-Deficient Human Cells

**Fig. 1:**
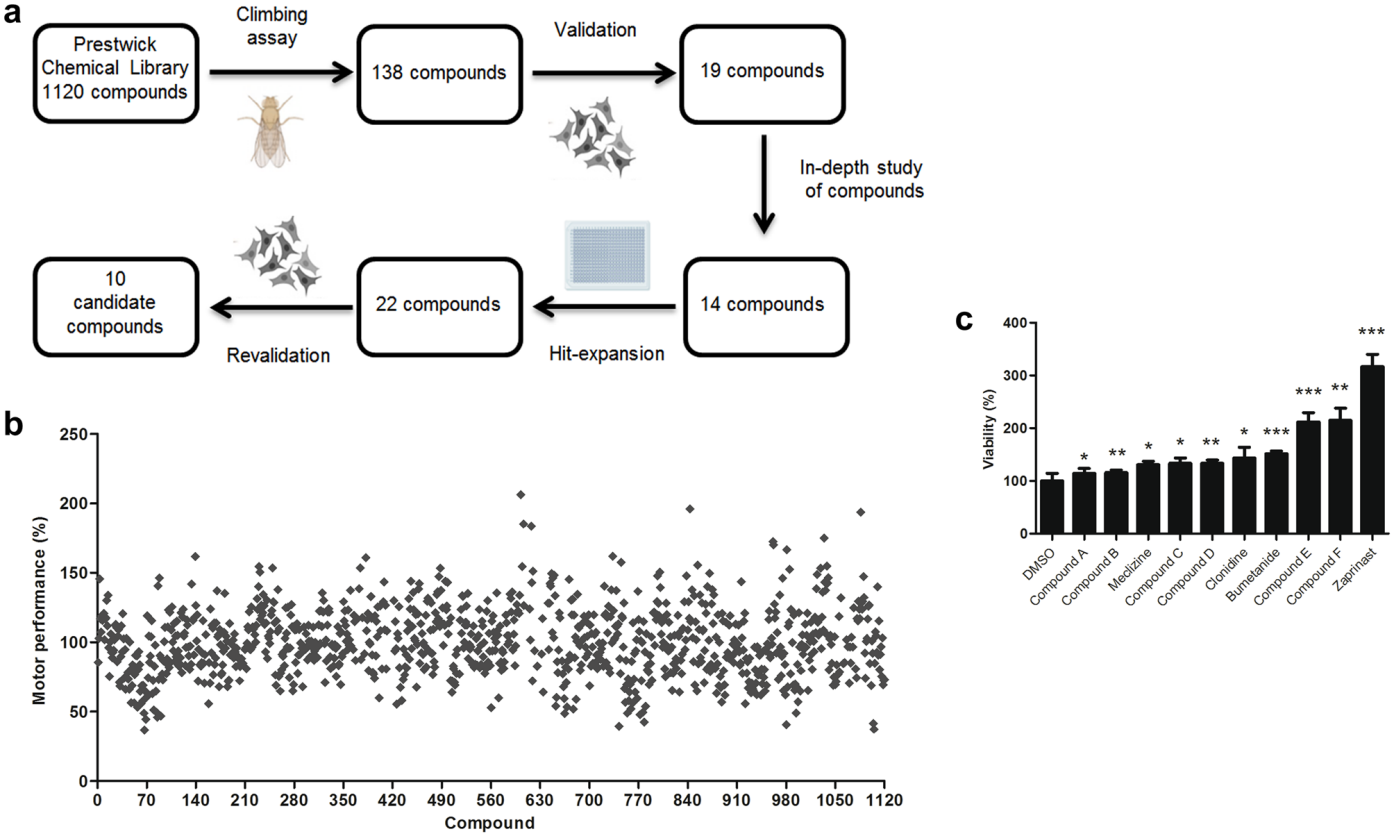
A High-throughput chemical screen and drug validation. **a** Scheme of the screening procedure used in the current study. Each screening stage (depicted by an arrow) led to several positive-hit compounds taken to the next stage. **b** Motor performance of *DJ-1ß* mutant flies obtained during the primary HTS for the 1120 evaluated drugs analyzed by climbing assays. Results are normalized to data obtained in *DJ-1ß* mutants treated with vehicle (DMSO). See “Material and Methods” section for details. **c** The viability of *DJ-1*-deficient cells was measured by MTT assays in the presence of OS (induced with 100 µM H_2_O_2_) and treated with the ten selected compounds. Results are normalized to data obtained in vehicle-treated *DJ-1*-deficient cells (DMSO). Error bars show s.d. from three independent biological replicates (*P < 0.05; **P < 0.01; ***P < 0.001)

HTS assays for drug discovery carried out in cell models do not account for anatomical integrated physiological factors that impact a drug’s ability to interact with its target or modify the response to target engagement. Results obtained using in vivo preclinical models provide a much more unambiguous indication of the potential pharmacological effects of a drug in humans [39, 40]. To identify novel treatments for PD, we performed an in vivo HTS assay using a *Drosophila* PD model (*DJ-1β* inactivation) and compounds from the PCL library, which are dissolved in 100% DMSO at a final concentration of 5 mM. The assay's rationale was that drugs able to suppress the motor deficits observed in *DJ-1β* mutant flies, a phenotype resembling a classic PD symptom [2], may also display benefits when tested in *DJ-1-*deficient cells [34]. Figure 1a provides an overview of the screening strategy used in this study.

First, we evaluated the effect of the 1120 PCL drugs on the locomotor ability of *DJ-1β* mutants by culturing L2 larvae in media supplemented with each compound at a concentration of 10 μM during development and 5 days after fly eclosion, and climbing assays were performed at that age (see “Material and Methods”). Among the drugs evaluated, 138 significantly improved the locomotor performance of *DJ-1β* mutant flies (Fig. 1a, b). We then evaluated the ability of these compounds to reduce OS-induced death in *DJ-1-*deficient SH-SY5Y cells [34]. We discovered that 19 of the 138 compounds identified in the screen performed in PD model flies displayed neuroprotective effects in *DJ-1*-deficient cells (validation step, Fig. 1a). We discarded 5 molecules after an in-depth literature review of their properties and adverse reactions given the description of Parkinsonism or motor dysfunctions among side effects in humans (Fig. 1A). At this point, we retested 8 drugs from the PCL not identified to be beneficial for PD model flies in the primary screen but with a mechanism of action similar to the 14 remaining drugs in the final list of potential therapeutic compounds (hit-expansion step, Fig. 1a). Finally, we carried out a revalidation step with the 22 leads in *DJ-1-*deficient cells and selected 10 candidate compounds that exhibited clear beneficial effects in cell viability assays (Fig. 1a, c).

Encouragingly, this list included two compounds (clonidine and bumetanide) currently under evaluation in Phase II clinical trials in PD patients (NCT03552068 and NCT03899324, respectively), which supports the relevance of results obtained in the HTS assay. We also identified meclizine, an FDA-approved antiemetic drug recently shown to attenuate PD-related phenotypes in fly and human cell models based on *DJ-1* inactivation by increasing glycolysis [22]. Other compounds belonged to different chemical categories (such as phosphodiesterase (PDE) inhibitors or corticosteroids) prescribed for various therapeutic indications (anti-hypertensive, anti-inflammatory, or antibiotic compounds). As zaprinast (ZAP) reduced OS-induced death in *DJ-1* mutant cells by the most significant degree (Fig. 1c), we selected this compound for further analyses. This drug is a phosphodiesterase (PDE) inhibitor, and can also activate the GPR35 orphan G-protein coupled receptor [41, 42]. Interestingly, it was previously reported that ZAP restores striatal long-term depression in mice transgenic for *A53T-SNCA* [43], and that it attenuated L-DOPA-induced dyskinesia in genetic and toxin-induced models of PD in mice and rats [44, 45] by inhibiting PDEs in both cases. Therefore, we assessed the effect of ZAP supplementation in our fly and human cell PD models to determine any disease-modifying potential.

### Zaprinast Suppresses PD-Related Phenotypes in *DJ-1ß* and *parkin* Mutant Flies

**Fig. 2:**
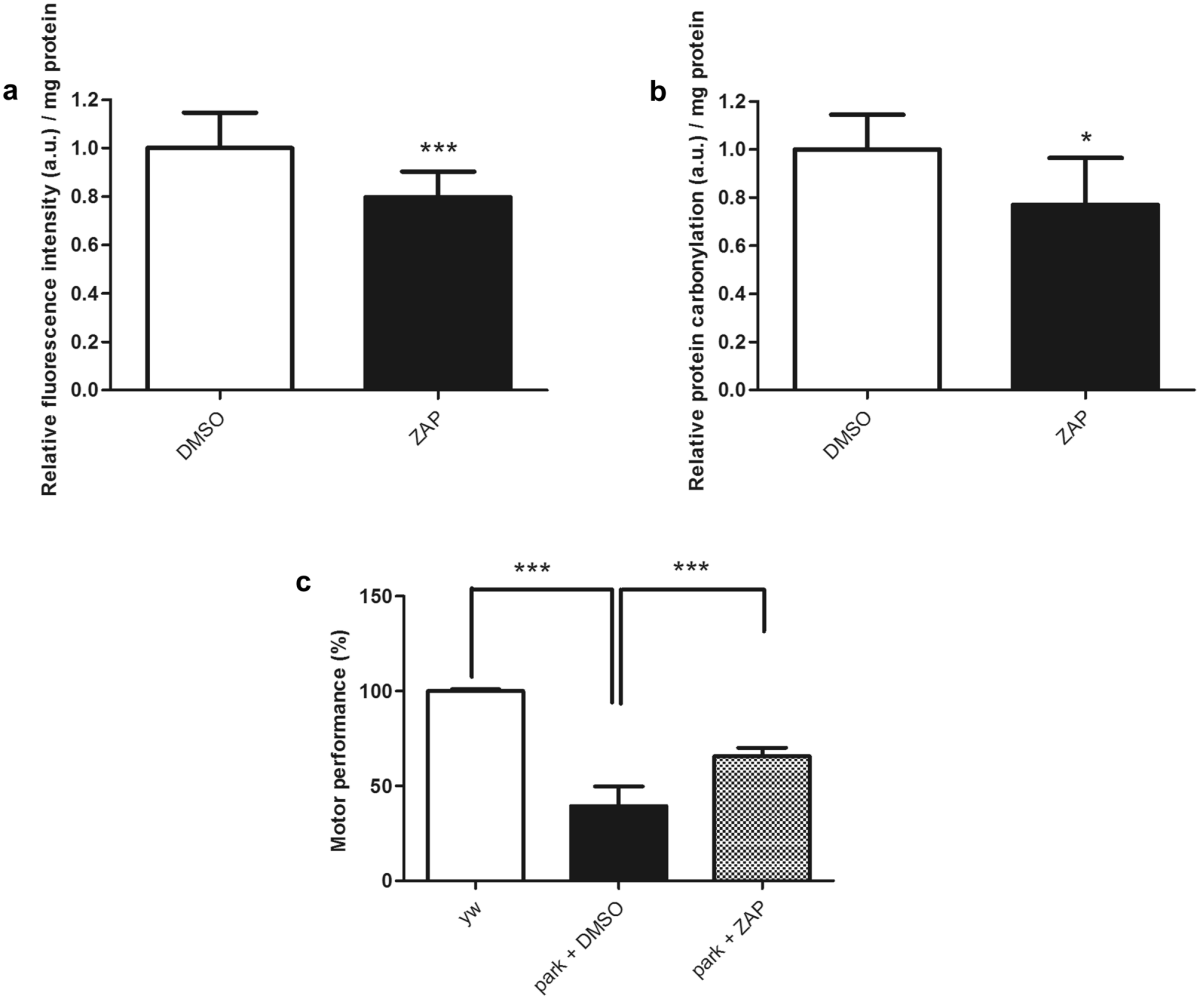
Effect of zaprinast on PD model flies. **a** H_2_O_2_ levels in *DJ-1β* mutant flies treated with 10 μM ZAP were analyzed using the Amplex H_2_O_2_ Red Kit (Invitrogen). **b** Protein carbonylation levels in *DJ-1ß* mutants treated with 10 μM ZAP were analyzed by absorbance. In all cases, data were expressed as arbitrary units (a.u.) per mg of protein, and results were referred to data obtained in flies cultured in vehicle medium (DMSO). **c** Motor performance of *y*,*w* control flies and *park* mutant flies treated with vehicle (DMSO) or 1 µM ZAP was evaluated performing a climbing assay. Error bars show s.d. from at least three replicates and three independent experiments (*P < 0.05; ***P < 0.001)

The DJ-1 protein plays an essential role in the defense against OS through several pathways [46]. Accordingly, PD models based on *DJ-1*-deficiency present high OS levels [32, 33, 47], which are clearly associated with PD pathology [3]. We previously reported that *DJ-1ß* mutant flies exhibited increased levels of reactive oxygen species (ROS) and protein carbonylation (a consequence of high ROS levels) compared to control flies of the same age [32–34, 47]. Thus, we evaluated whether ZAP supplementation suppressed those phenotypes in *DJ-1β* mutant flies. Our results revealed that *DJ-1ß* mutants treated with ZAP during development and 5 days after eclosion presented a mild but significant reduction in H_2_O_2_ production (a component of the total ROS pool) compared to flies treated with vehicle (Fig. 2a). Consistently, we also observed a significant reduction in protein carbonylation after ZAP supplementation (Fig. 2b). Therefore, ZAP treatment in PD model flies based on *DJ-1β* deficiency was able to reduce OS levels, which have been shown to have a causative role in their motor deficits [34]. Of note, our preliminary results established that ZAP is also able to suppress motor defects in *park* mutant flies (Fig. 2c), another *Drosophila* model of familial PD [38]. Considering that no ortholog of human GPR35 does exist in the *Drosophila* genome, ZAP is probably exerting its beneficial effect in PD model flies through its PDE inhibitor activity. Indeed, *Drosophila* PDE1, PDE6, and PDE11 function were already shown to be sensitive to ZAP treatment [48]

### Zaprinast Activates Akt Signaling and Downregulates the JNK Pathway in *DJ-1*-deficient human cells

**Fig. 3:**
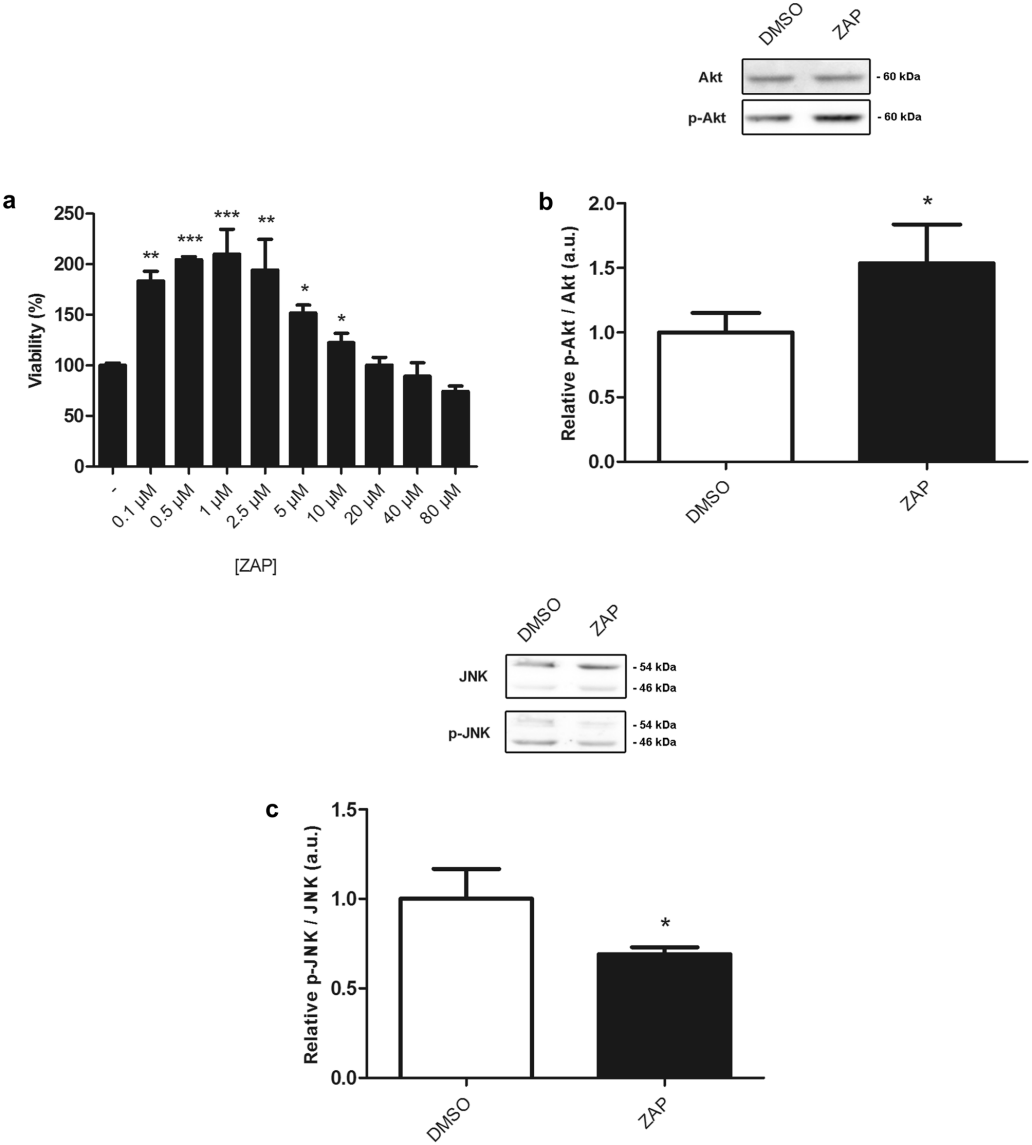
Effect of zaprinast on viability and Akt/JNK pathway activities in *DJ-1-*deficient cells. **a** MTT assays measured the viability of *DJ-1*-deficient cells in the presence of OS (induced with 100 µM H_2_O_2_). Cells were either treated with vehicle (DMSO) or with ZAP (0.1–80 μM). Results were normalized to data obtained in vehicle-treated mutant cells (-). **b**, **c** Antibodies against Akt, p-Akt, JNK, and p-JNK were used to detect proteins of interest in *DJ-1*-deficient cells subjected to OS and treated with 1 μM ZAP by Western blot (upper panels). The relative ratios of p-Akt/Akt and p-JNK/JNK were analyzed by densitometry (lower panels). Results are referred to data obtained in vehicle-treated *DJ-1*-deficient cells and expressed as arbitrary units (a.u.). In all cases, error bars show s.d. from three independent experiments in which three biological replicates were used (*P < 0.05; ***P < 0.001)

We next determined any neuroprotective effect of ZAP in *DJ-1*-deficient cells (as shown in Fig. 1c). First, we evaluated the ability of ZAP exposure to protect against the OS-induced death of *DJ-1-*deficient human neuroblastoma cells in a dose-dependent manner by pretreating them with ZAP (0.1–80 µM) and measuring viability via MTT assays. Our results demonstrated that ZAP significantly attenuated cell death at low concentrations (between 0.1 and 10 µM), with 1 µM the most effective (Fig. 3a). As shown in Fig. S1, ZAP did not affect viability in control cells at those concentrations.

Apoptosis has been identified as an important mechanism that leads to neuronal death in PD [49]. This process is highly regulated by the activity of several kinases like the pro-survival factor Akt and the pro-apoptotic factor JNK [50]. Previous studies revealed that *DJ-1* knockdown in human neuroblastoma SH-SY5Y cells and rat adrenal pheochromocytoma PC12 cells decreased Akt phosphorylation, thus suppressing the Akt signaling pathway. Besides, it also increased JNK phosphorylation, thus overactivating JNK signaling pathway and promoting cell death [51, 52]. Analysis of Akt and JNK phosphorylation levels in our *DJ-1-*deficient cells confirmed the presence of similar molecular alterations (Fig. S2). Since ZAP showed a neuroprotective effect in *DJ-1-*deficient cells, we performed Western blot assays to determine whether pretreatments with 1 µM ZAP affected Akt or JNK phosphorylation levels in such cells. Phosphorylation of Akt at Ser473 and total Akt levels were measured in mutant cells pretreated with ZAP. Our results showed that ZAP supplementation was able to significantly increase Akt phosphorylation (Fig. 3b), which leads to its activation [51]. Besides, mutant cells pretreated with ZAP also displayed a significant reduction of JNK phosphorylation at Thr183 and Tyr185 (Fig. 3c), leading to its inactivation [51].

Taken together, these results support the neuroprotective effect of ZAP supplementation in *DJ-1-*deficient cells through the modification of Akt and JNK pathway activation. Previous studies reported that several PDE inhibitors exhibited anti-apoptotic properties through Akt pathway activation [53, 54]. Furthermore, neuroprotection mediated by pamoic acid, a potent GPR35 agonist, in stroke associates with an increase in Akt phosphorylation [55]; therefore, ZAP may exert its neuroprotective effect in PD model cells based on *DJ-1* deficiency either by inhibiting PDEs or by activating GPR35.

### Zaprinast Increases Mitochondrial Viability in *DJ-1*-Deficient Human Cells

**Fig. 4:**
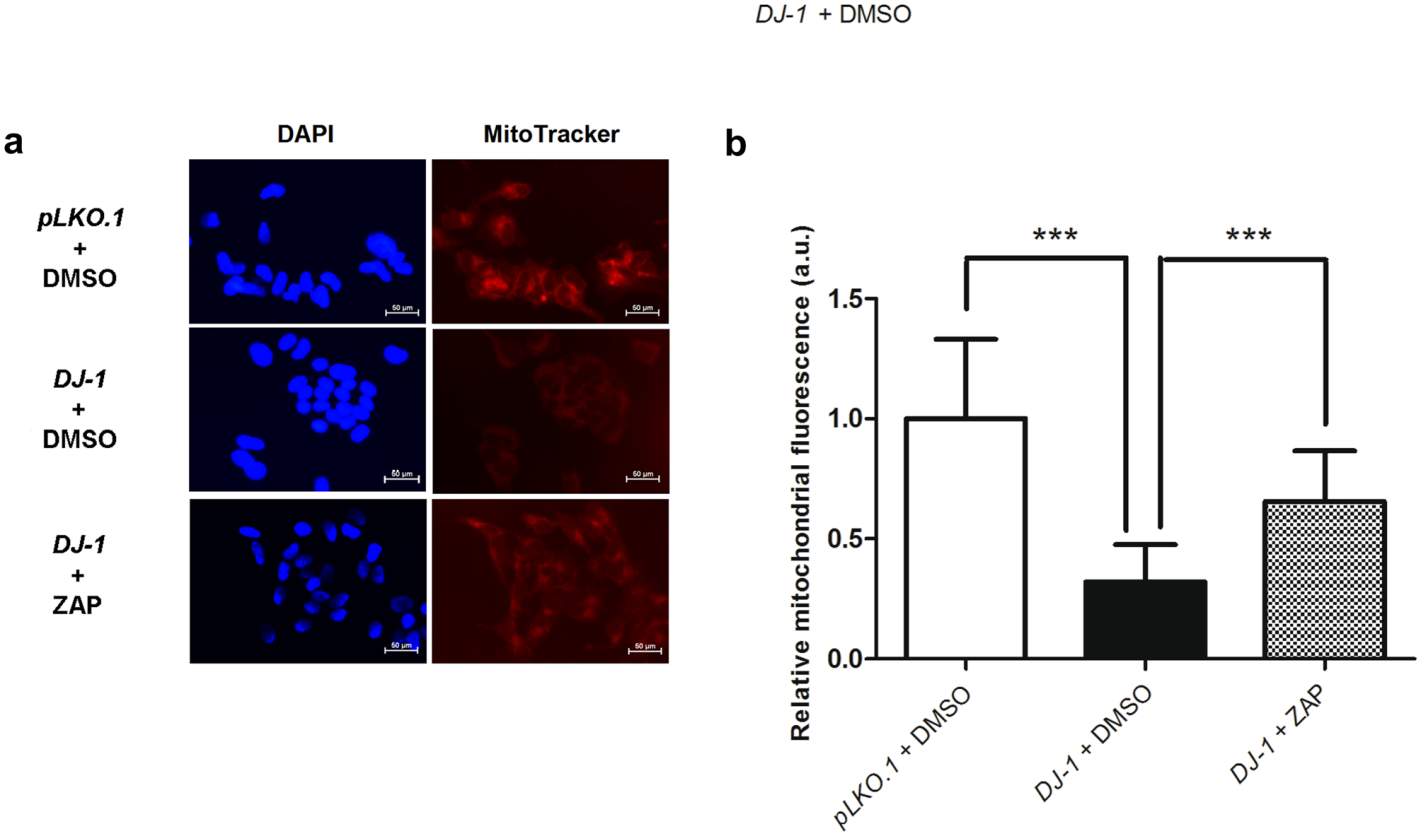
Effect of zaprinast on mitochondrial activity in *DJ-1*-deficient cells. **a** Representative images of SH-SY5Y cells stained with MitoTracker Red FM, a specific mitochondrial dye, and the nuclear dye DAPI (blue) acquired via fluorescence microscopy. Cells stained were *pLKO.1* control cells and *DJ-1*-deficient cells pretreated with vehicle (DMSO), and *DJ-1*-deficient cells treated with 1 µM ZAP. Scale bar, 50 µm. **b** Graphical representation of Mitotracker Red FM fluorescence quantification from **a**. At least ten images of each strain and condition were analyzed. Results are normalized to data obtained in vehicle-treated *pLKO.1* control cells and expressed as arbitrary units (a.u.). Error bars show s.d. from three independent experiments in which three biological replicates were used (***P < 0.001)

Mitochondrial dysfunction has been related to PD pathogenesis [56]. Accordingly, previous studies have revealed that *DJ-1* knockdown decreases active mitochondrial mass and alters mitochondrial morphology and function [51, 57, 58]. Mitochondrial alterations in *DJ-1-*deficient cells could be associated with the inhibition of Akt pathway and with JNK activation [51, 59], which have both been proposed as novel therapeutic targets for PD [51]. As ZAP supplementation led to Akt activation and JNK inhibition (Fig. 3b, c), we hypothesized that these changes might improve mitochondrial viability. We discovered that *DJ-1* mutant cells displayed a significant decrease in the active mitochondrial mass compared to *pLKO.1* control cells using the MitoTracker™ Red FM dye, as evidenced by a reduction in fluorescence intensity (Fig. 4). As expected, pretreatment of *DJ-1-*deficient cells with ZAP resulted in a significant increase in the number of viable mitochondria compared to vehicle-treated cells (Fig. 4), supporting the therapeutic potential of ZAP as a means to ameliorate PD-associated mitochondrial dysfunction.

### Zaprinast Enhances the Activity of Key Glycolytic Enzymes in *DJ-1*-Deficient Human Cells

**Fig. 5:**
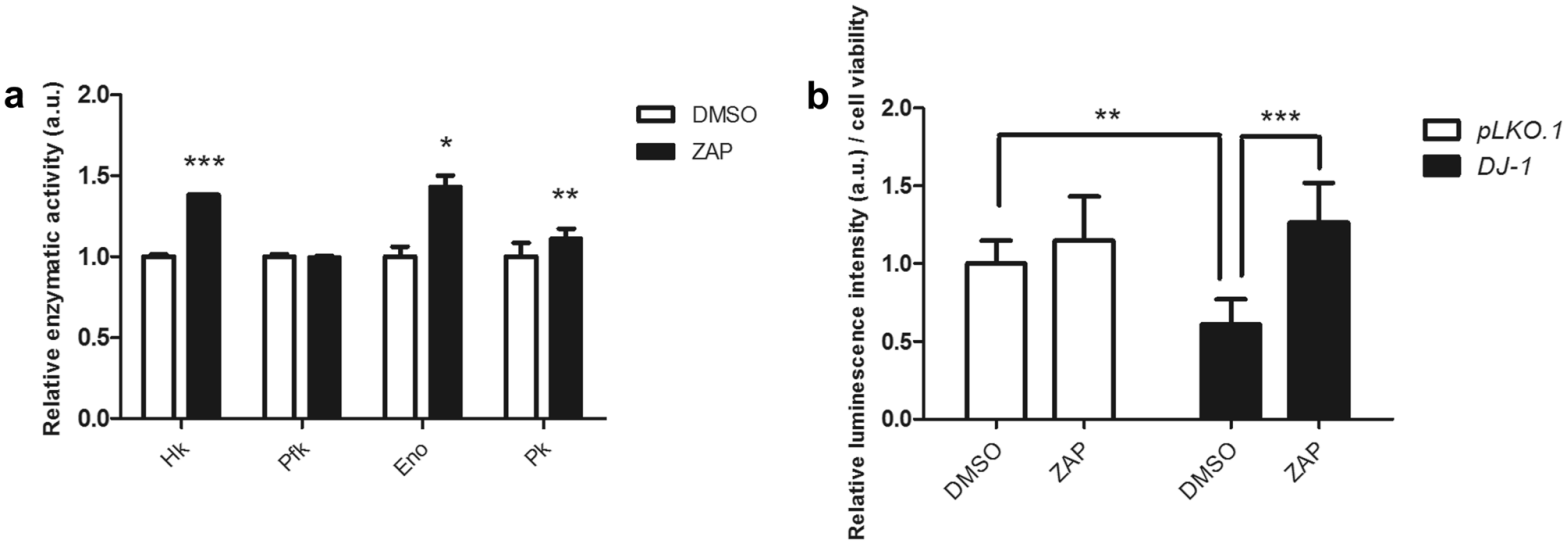
Effect of zaprinast on the activity of glycolytic enzymes and on ATP levels in *DJ-1*-deficient cells. **a** The activity of hexokinase (Hk), phosphofructokinase (Pfk), enolase (Eno), and pyruvate kinase (Pk) in *DJ-1*-deficient cells treated with 1 µM ZAP under OS condition induced with 50 µM H_2_O_2_. Results were normalized to data obtained in vehicle-treated cells (DMSO). In all cases, error bars show s.d. from three replicates and three independent experiments (*P < 0.05; **P < 0.01; ***P < 0.001). **b** ATP levels in *DJ-1*-deficient cells in the presence of OS (induced with 50 µM H_2_O_2_) treated with vehicle (DMSO) or with 1 μM ZAP were analyzed using the ATP Determination Kit (Invitrogen). Results were normalized to data obtained in vehicle-treated *pLKO.1* cells (DMSO). Error bars show s.d. from three independent experiments in which three biological replicates were used (**P < 0.01; ***P < 0.001)

It has been recently shown that PD is also characterized by metabolic alterations [22, 60, 61]. In fact, lack of *DJ-1* leads to an enhancement of the glycolytic pathway, probably as a means to counteract the reduction of ATP levels caused by mitochondrial dysfunction [22, 62]. Despite this, it has been reported that primary midbrain cultures from *DJ-1* mice embryos still showed reduced ATP levels when compared to wild-type controls [63]. The glycolytic rate can be evaluated by measuring the activity of key enzymes involved in this pathway [64], and we recently reported that *DJ-1*-deficient cells presented higher activities of hexokinase (Hk), phosphofructokinase (Pfk), enolase (Eno), and pyruvate kinase (Pk) when compared to vehicle-treated cells [22]. Furthermore, several studies have demonstrated that enhancing glycolysis may represent a promising target for PD treatment [22, 65]. Therefore, we evaluated whether ZAP treatment affected glycolysis in *DJ-1*-deficient cells by quantifying the activity of these key glycolytic enzymes. Interestingly, our results demonstrated that ZAP supplementation led to a significant and robust increase in Hk and Eno activity, and to a mild increase of Pk activity in *DJ-1*-deficient cells (Fig. 5A). According to this, we found that this enhancement of glycolysis resulted in an increase in ATP levels in ZAP-treated *DJ-1* mutant cells compared to those treated with vehicle (Fig. 5b). These results confirm our previous hypothesis that the increase in the glycolytic pathway is aimed to recover ATP levels [22] which are reduced in *DJ-1*-deficient cells when compared to controls (Fig. 5b). Our results also showed that ZAP treatment led to increase Eno and Pfk activities in control cells (Fig. S3), although no significant changes in ATP levels were found in such cells (Fig. 5b). Taken together, these results indicate that ZAP may contribute to enhance glycolysis and to restore ATP levels in PD model cells. A previous study had suggested that inhibition of cGMP PDE isoforms may control the ability of ZAP to increase insulin-mediated glucose uptake in skeletal muscles [66]; moreover, GPR35 has been also shown to promote glycolysis by activating Na/K-ATPase activity [67].

### The GPR35 Antagonist CID2745687 Diminishes the Neuroprotective Effect of Zaprinast in *DJ-1*-Deficient Human Cells

**Fig. 6:**
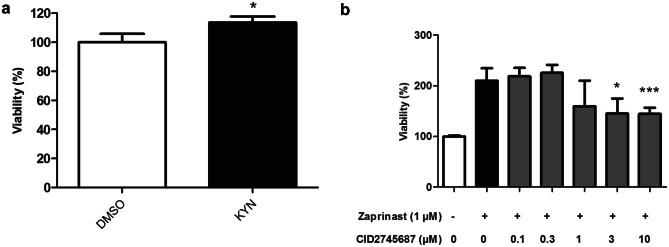
Effect of kynurenic acid and CID2745687 in *DJ-1*-deficient cells. **a** MTT assays measured the viability of *DJ-1*-deficient cells in the presence of OS (induced by 100 µM H_2_O_2_). Cells were either treated with vehicle (DMSO) or with 10 µM kynurenic acid. **b** MTT assays measured the viability of *DJ-1*-deficient cells in the presence of OS (induced with 100 µM H_2_O_2_) treated with 1 µM ZAP. Cells were either pretreated with different concentrations of CID2745687 (0.1–10 µM) or with vehicle (-). Results were normalized to data obtained in vehicle-treated mutant cells. Error bars show s.d. from three independent biological replicates (*P < 0.05; ***P < 0.001)

As previously mentioned, ZAP is a PDE inhibitor but also a GPR35 agonist. While PDE inhibitors have been already proposed as possible PD therapies [68], very little is known about the effect of GPR35 agonists in PD models. Therefore, we decided to evaluate whether GPR35 activation could exert any neuroprotective effect in our human cell PD model. First, we confirmed by RT-qPCR analyses that the *GPR35* gene was mildly expressed in SH-SY5Y cells (data not shown), as reported in [69]. Subsequently, we tested whether supplementation with kynurenine (KYN) could affect the viability of *DJ-1-*deficient cells. KYN is the precursor of kynurenic acid (KYNA) [70], which may represent the endogenous GPR35 ligand [71]. Consistently, our results showed that KYN treatment displayed a mild but significant neuroprotective effect in *DJ-1-*deficient cells (Fig. 6a), thus indicating that GPR35 agonists may represent potential candidates for PD treatment. Therefore, we hypothesized that ZAP could exert its neuroprotective effect in PD model cells through GPR35 activation. To confirm this assumption, we tested whether CID2745687 (methyl-5-[(tert-utylcarbamothioylhydrazinylidene)methyl]-1-(2,4-difluorophenyl)pyrazole-4-carboxylate), the only well-characterized GPR35 antagonist [72], could interfere with ZAP-mediated neuroprotection. We found that viability of ZAP-treated *DJ-1*-deficient cells was significantly reduced when pretreated with increasing concentrations of CID2745687 (Fig. 6b). In contrast, viability was not affected by CID2745687 treatment in *DJ-1*-deficient or control cells (Fig. S4). In summary, our results indicate that ZAP is able to reduce degeneration of PD model cells in part through GPR35 activation thus suggesting that GPR35 agonists could be considered as possible PD therapies.

## Discussion

As PD remains an incurable disease and the fastest growing neurological disorder globally [73], we urgently need to develop novel, effective therapeutic strategies. With this aim, we performed an HTS assay using an in vivo *Drosophila* PD model to evaluate the effect of a range of compounds on a behavioral phenotype. To our knowledge, this study represents the first HTS assay carried out in adult flies that evaluates motor performance. We screened the PCL, a library that contains 1120 compounds most of them approved by regulatory agencies (FDA and EMA), and tested for bioavailability and safety in humans. Hence, our results may allow drug repurposing, a new trend in drug discovery by which new therapeutic applications for an existing drug are found, accelerating their potential for use in PD patients [74]. We identified ten compounds that improved locomotor activity in *DJ-1ß* mutant flies and increased viability in *DJ-1*-deficient cells subjected to OS. We focused on the non-commercialized drug ZAP as the most efficient candidate compound regarding improvements to the viability of PD model cells. We also identified compounds that reduced motor defects in *DJ-1β* mutant flies but had no effect on the viability of *DJ-1*-deficient cells during the validation step. While some could represent false positives, others could target other PD-relevant cell types, such as glial cells [75]; therefore, different validation assays with additional PD model cells may confirm their therapeutic potential.

ZAP is an inhibitor of the PDE1, 5, 6, 9, and 11 isoforms and a GPR35 agonist [41, 42], and studies have highlighted the expression of both PDE1 and GPR35 in the midbrain [68, 69]. PDE inhibitors have been proposed as PD therapies, evaluated in many preclinical studies, and patented as possible anti-PD drugs [68]. These compounds act by repressing the degradation of cAMP and/or cGMP, cyclic nucleotides involved in many CNS processes [68, 76]. Interestingly, alterations in cAMP and cGMP synthesis/degradation may lead to the onset of age-related diseases, including PD [76]. One of the multiple functions of both cyclic nucleotides is the regulation of the apoptosis-related Akt and JNK signaling pathways [53, 68, 77]. Here, we report that ZAP exerted a beneficial function in *DJ-1-*deficient SH-SY5Y cells by reducing stress-induced apoptosis through the activation of Akt and inhibition of JNK signaling [51, 52]. Therefore, these results agree with the function of ZAP as a PDE inhibitor. Indeed, its beneficial effect in PD model flies is probably exerted by this mechanism since no GPR35 orthologs are present in the *Drosophila* genome.

In contrast, we know relatively little about the GPR35 orphan receptor and its relation to PD, and ZAP could be acting not only as a PDE inhibitor but also as a GPR35 activator in our PD model human cells Studies have suggested a potential role for GPR35 in regulating neuronal excitability and synaptic release [78, 79] and controlling inflammation and the immune system [72, 80]. ZAP also inhibits N-type calcium channels through GPR35 activation [81], which have been found hyperactivated and overexpressed in an *α-syn* mouse model and linked to axonal degeneration [82]. These findings along with our results with ZAP led us to hypothesize that the pharmacological activation of GPR35 could represent a novel therapeutic approach to PD. Supporting this assumption, we found that KYN, the precursor of the endogenous GPR35 ligand KYNA [71], displayed a mild but significant neuroprotective effect in *DJ-1-*deficient cells. Consistently, we found that pretreatment of PD model cells with the bona fide GPR35 antagonist, CID2745687, was able to partially abolish the neuroprotective effect of ZAP in such cells. Taken together, these results indicate that GPR35 agonists may represent potential candidates for PD treatment. Interestingly, studies have demonstrated alterations to the KYN pathway (a reduction of KYNA levels) in PD patients with L-DOPA-induced dyskinesia [83]. Furthermore, while endogenous KYNA exhibits neuroprotective activity, its metabolites present neurotoxicity [84]; therefore, the KYN pathway may represent a promising target in the search for PD treatments. Accordingly, the inhibition of enzymes related to the degradation of KYNA also inhibits neurodegeneration in fly models of diseases, including PD [85].

This study also demonstrated that ZAP exerted its beneficial effect in PD models through different mechanisms. Mitochondrial alterations and high OS levels play an important role in PD development as well as in other neurodegenerative diseases [86, 87] and are strongly related to energy metabolism [60, 88]. Recent studies have highlighted the role of metabolic alterations in PD [22, 60, 61], with many genes involved in familial PD functionally linked to mitochondria (e.g., *DJ-1*, *PRKN*, or *PINK1*) [56]. Mitochondrial dysfunction leads to increased OS levels and reduced ATP production in PD models, which are counteracted by an increased glycolytic rate [22, 89, 90]. Interestingly, increasing glycolysis has been recently described as a potential therapeutic strategy for PD [22, 65]. Here, we confirmed the reduction in mitochondrial viability in *DJ-1-*deficient cells [51], which may increase OS accompanied by alterations in energy metabolism [88]. We demonstrated that ZAP supplementation increases mitochondrial viability in *DJ-1*-deficient cells, which in turn would cause a reduction in OS levels [90]. Furthermore, *DJ-1*-deficient cells pretreated with ZAP also displayed enhanced glycolysis, which led to an increase in ATP levels. As a result, ZAP exerted a protective effect in fly and human cell PD models based on *DJ-1* deficiency by intervening in several cellular alterations with a pivotal role in PD.

Despite current efforts to encounter novel treatments for PD and the proposal of a considerable amount of compounds as potential therapeutics [29, 91], PD remains an incurable disease [73]. Candidate compounds obtained in preclinical models often fail when evaluated in clinical trials due to various factors, such as disease and patient heterogeneity, inadequate trial design, inappropriate endpoints, and poor patient selection. Our knowledge regarding PD physiopathology also remains incomplete [91, 92], thereby contributing to the selection of non-optimal candidate compounds in preclinical studies. For this reason, the identification of compounds that target multiple phenotypes in several disease models may allow for improved drug discovery in neurodegenerative diseases [36]. This study demonstrated that ZAP exerts its beneficial function in PD models through different disease-modifying mechanisms in PD models based on *DJ-1* dysfunction. Given the discovery of over-oxidized and inactive DJ-1 protein in sporadic PD patients [23], we hypothesize that therapeutic compounds identified in *DJ-1* models could find use in individuals with sporadic PD.

In summary, we identify ZAP as a potential therapeutic compound for PD using an HTS assay in a *Drosophila* model. PD is a multifactorial disorder [93]; therefore, a multi-therapy approach may be required to treat PD patients efficiently. In this sense, ZAP represents a promising drug, given its function through both PDEs and GPR35 [41, 42], thus widening the therapeutic landscape. Although our results clearly demonstrate that the neuroprotective effect of ZAP in PD model cells is reduced by pretreatments with the GPR35 antagonist CID2745687, further studies are required to validate GPR35 agonists as novel therapies for PD. This study also validates *Drosophila* as a valuable model organism with huge potential in the drug discovery field, which may lead to the identification of novel therapies for PD and other human diseases.

## Supplementary Information

Below is the link to the electronic supplementary material.

Supplementary file1 (PDF 1383 kb)

Supplementary file2 (PDF 1391 kb)

Supplementary file3 (PDF 1381 kb)

Supplementary file4 (PDF 1376 kb)

Supplementary file5 (PDF 1381 kb)

Supplementary file6 (PDF 1387 kb)

Supplementary file7 (DOCX 256 kb)

FigS1 (TIF 861 kb)

FigS2A (TIF 4 MB)

FigS2B (TIF 4 MB)

FigS3 (TIF 273 kb)

FigS4A (TIF 875 kb)

FigS4B (TIF 877 kb)
