# Supplementary material for: A High-Throughput Chemical Screen in DJ-1β Mutant Flies Identifies Zaprinast as a Potential Parkinson’s Disease Treatment
Source: Neurotherapeutics. 2021 Oct 25;18(4):2565–78. doi: 10.1007/s13311-021-01134-2 (PMC8804136; doi:10.1007/s13311-021-01134-2)
Supplement: Supplementary file 7 — Supplementary file7 (DOCX 256 kb) [file 13311_2021_1134_MOESM7_ESM.docx]

**SUPPLEMENTARY FIGURES**

**Fig. S1** Effect of zaprinast on viability in *pLKO.1* cells. MTT assays measured the viability of *DJ-1*-deficient cells in the presence of OS (induced with 100 µM H_2_O_2_). Cells were either treated with vehicle (-) or with ZAP (0.1-80 μM). Results were normalized to data obtained in vehicle-treated cells. Error bars show s.d. from three independent biological replicates (*, P < 0.05).

**Fig. S2** Akt and JNK phosphorylation levels in *DJ-1-*deficient cells. (A) p-Akt/Akt and (B) p-JNK/JNK protein levels were detected in *DJ-1*-deficient cells and *pLKO.1* control cells in the presence of OS (induced with 100 μM H_2_O_2_) by Western blot analyses (upper panels). The relative ratios of p-Akt/Akt and p-JNK/JNK were analyzed by densitometry (lower panels). Results are normalized to data obtained in *pLKO.1* control cells in the presence of OS and are expressed as arbitrary units (a.u.). Error bars show s.d. from three independent experiments in which three biological replicates were used (*, P < 0.05; **, P < 0.01).

**Fig. S3** Effect of zaprinast on the activity of glycolytic enzymes in *pLKO.1* control cells. The activity of hexokinase (Hk), phosphofructokinase (Pfk), enolase (Eno), and pyruvate kinase (Pk) in control cells treated with 1 µM ZAP under OS condition induced with 50 µM H_2_O_2_. Results were normalized to data obtained in vehicle-treated cells (DMSO). In all cases, error bars show s.d. from three replicates and three independent experiments (***, P < 0.001).

**Fig. S4** Effect of CID2745687 on viability assays in cells. (A) MTT assays measured the viability of control cells treated with vehicle (-) or with CID2745687 (0.1-10 μM) (B) MTT assays measured the viability of *DJ-1*-deficient cells in the presence of OS (induced with 100 µM H_2_O_2_) treated with vehicle (-) or with CID2745687 (0.1-10 μM). Results were normalized to data obtained in vehicle-treated cells. Error bars show s.d. from three independent biological replicates.
