# Supplementary figures and images for: A High-Throughput Chemical Screen in DJ-1β Mutant Flies Identifies Zaprinast as a Potential Parkinson’s Disease Treatment
Source: Neurotherapeutics. 2021 Oct 25;18(4):2565–78. doi: 10.1007/s13311-021-01134-2 (PMC8804136; doi:10.1007/s13311-021-01134-2)

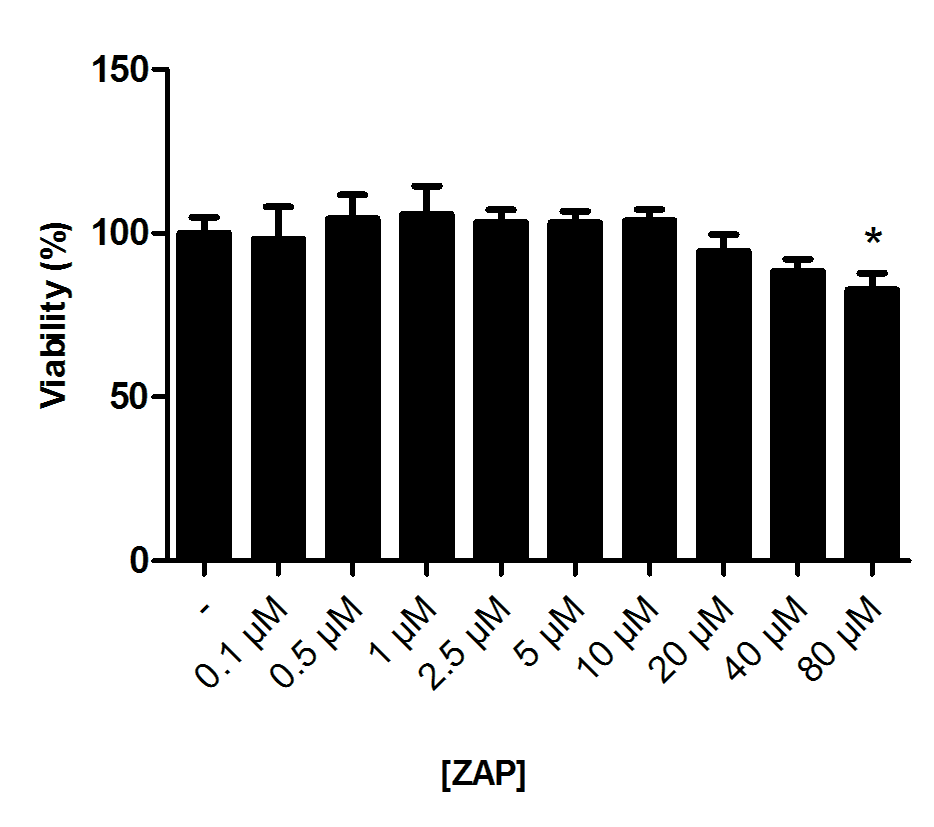

Supplement: Supplementary file 8 — FigS1 (TIF 861 kb) [file 13311_2021_1134_MOESM8_ESM.tif]

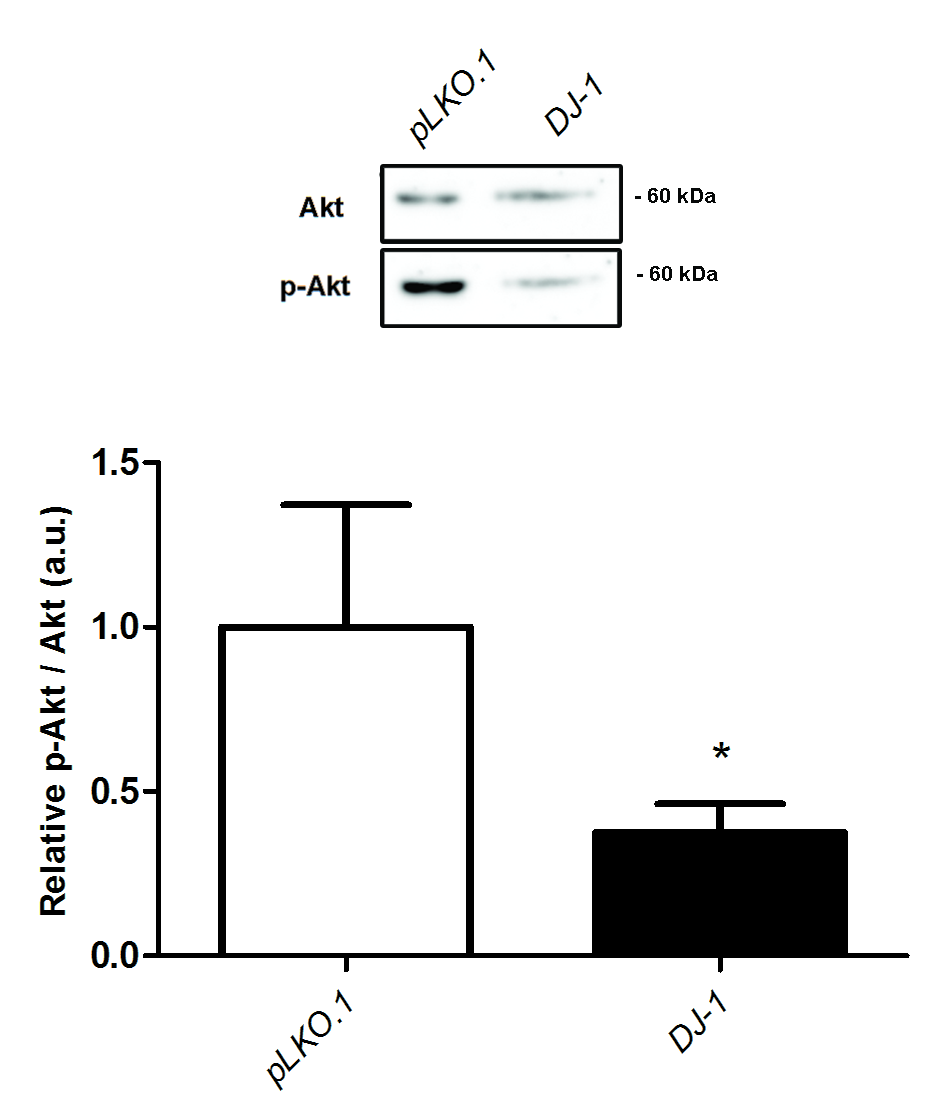

Supplement: Supplementary file 9 — FigS2A (TIF 4 MB) [file 13311_2021_1134_MOESM9_ESM.tif]

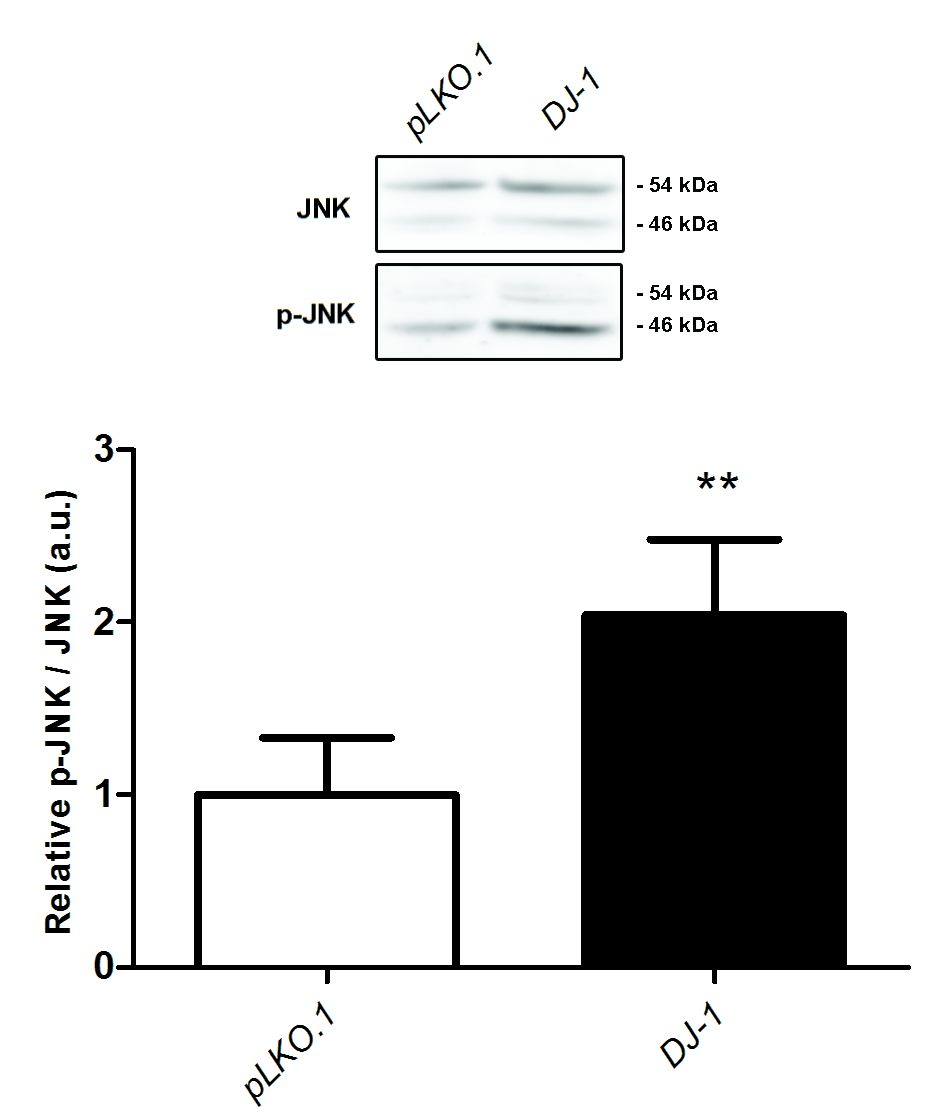

Supplement: Supplementary file 10 — FigS2B (TIF 4 MB) [file 13311_2021_1134_MOESM10_ESM.tif]

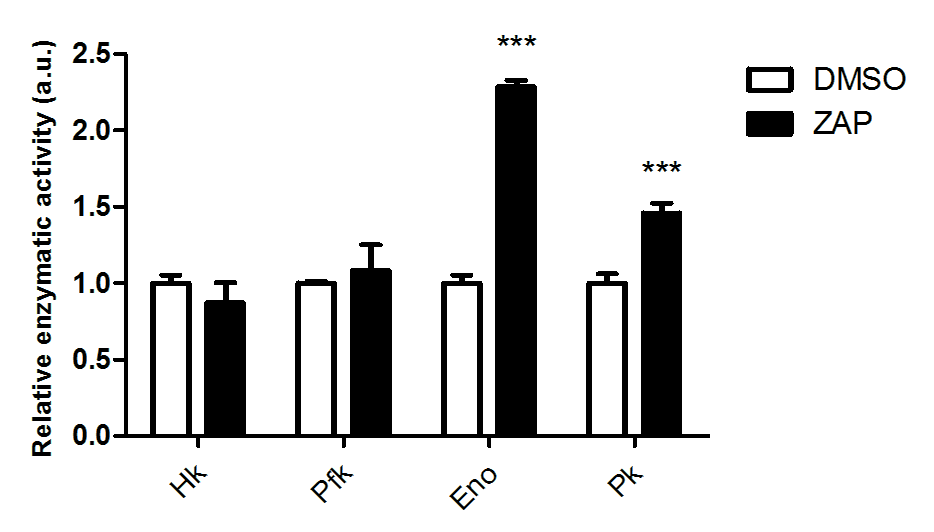

Supplement: Supplementary file 11 — FigS3 (TIF 273 kb) [file 13311_2021_1134_MOESM11_ESM.tif]

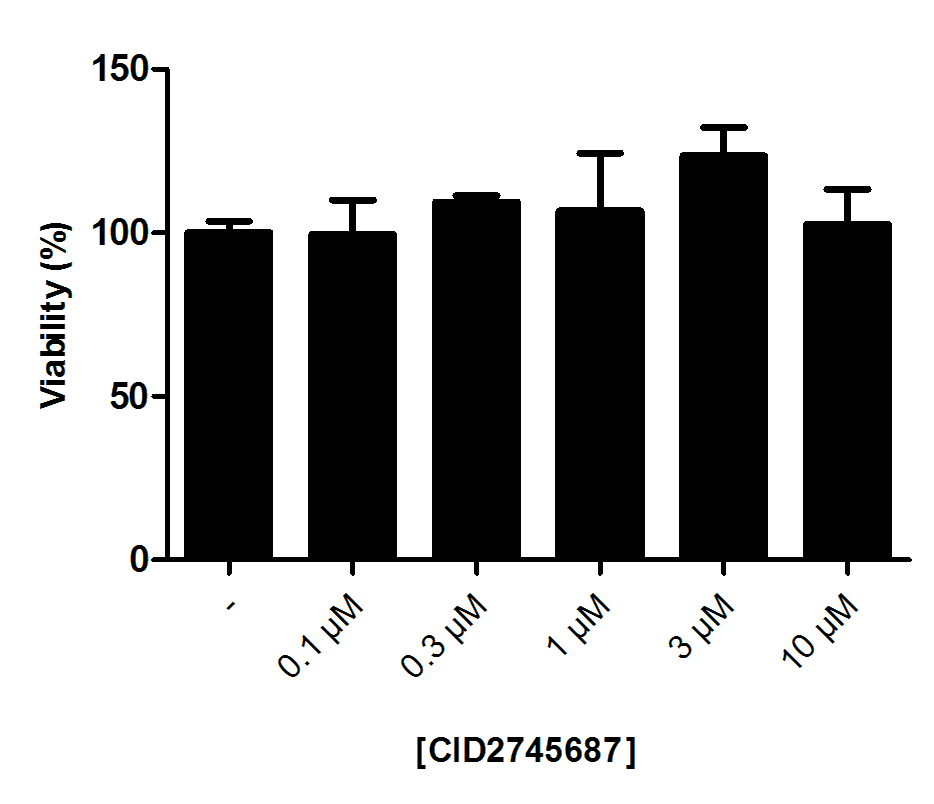

Supplement: Supplementary file 12 — FigS4A (TIF 875 kb) [file 13311_2021_1134_MOESM12_ESM.tif]

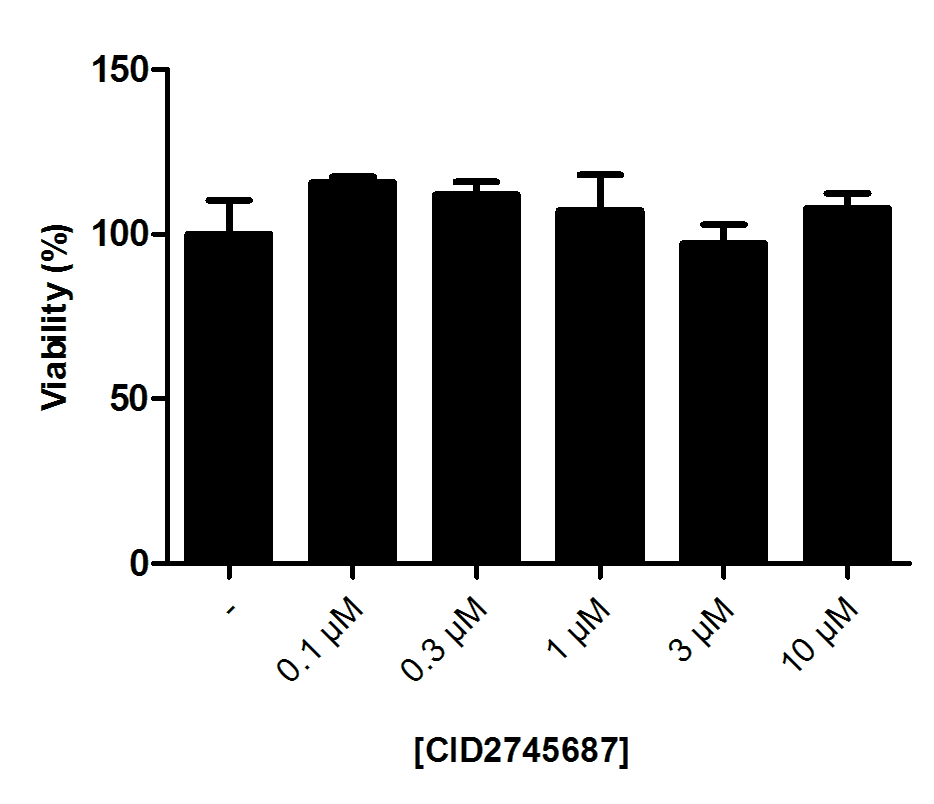

Supplement: Supplementary file 13 — FigS4B (TIF 877 kb) [file 13311_2021_1134_MOESM13_ESM.tif]
